# Quantifying cadherin mechanotransduction machinery assembly/disassembly dynamics using fluorescence covariance analysis

**DOI:** 10.1038/srep28822

**Published:** 2016-06-30

**Authors:** Pavan Vedula, Lissette A. Cruz, Natasha Gutierrez, Justin Davis, Brian Ayee, Rachel Abramczyk, Alexis J. Rodriguez

**Affiliations:** 1 Department of Biological Sciences, Rutgers, The State University of New Jersey, Newark, New Jersey, USA

## Abstract

Quantifying multi-molecular complex assembly in specific cytoplasmic compartments is crucial to understand how cells use assembly/disassembly of these complexes to control function. Currently, biophysical methods like Fluorescence Resonance Energy Transfer and Fluorescence Correlation Spectroscopy provide quantitative measurements of direct protein-protein interactions, while traditional biochemical approaches such as sub-cellular fractionation and immunoprecipitation remain the main approaches used to study multi-protein complex assembly/disassembly dynamics. In this article, we validate and quantify multi-protein adherens junction complex assembly *in situ* using light microscopy and Fluorescence Covariance Analysis. Utilizing specific fluorescently-labeled protein pairs, we quantified various stages of adherens junction complex assembly, the multiprotein complex regulating epithelial tissue structure and function following *de novo* cell-cell contact. We demonstrate: minimal cadherin-catenin complex assembly in the perinuclear cytoplasm and subsequent localization to the cell-cell contact zone, assembly of adherens junction complexes, acto-myosin tension-mediated anchoring, and adherens junction maturation following *de novo* cell-cell contact. Finally applying Fluorescence Covariance Analysis in live cells expressing fluorescently tagged adherens junction complex proteins, we also quantified adherens junction complex assembly dynamics during epithelial monolayer formation.

Fluorescence signal co-localization is widely used to assess protein complex assembly^1^. A number of global statistical methods, involving pixel intensity distributions, provide analysis options that are used to quantify co-localization^2^. Two such techniques, *M*anders *O*verlap *C*oefficient (MOC) and *M*anders *C*olocalization *C*oefficients (MCC)^3^ were developed to assess the degree of co-occurrence or the relative fraction of overlap of two fluorescence signals, respectively. However, both MOC and MCC analyses are unable to differentiate between signals from bona fide protein complexes and non-specific overlapping signals due to the large voxel size relative to the volume occupied by individual proteins. In addition, these coefficients are significantly affected by threshold corrections and require numerous processing steps to assign valid background values. Such corrections are especially difficult when working with low intensity signals, such as during early stages of multi-protein complex assembly^4^. Importantly, many multi-protein complexes maintain strict stoichiometry during their assembly, meaning individual components vary proportionally to other complex components. Consequently, fluorescence signals from proteins that are components of bona fide multi-protein complexes will not only co-occur within an individual pixel but they will also co-vary. In 1886, Karl Pearson introduced a correlation coefficient to study the extent to which two variables co-vary with each other^5^. The coefficient, termed *P*earson’s *C*orrelation *C*oefficient (PCC), is as a pixel-by-pixel covariance analysis method. Consequently, Pearson’s Correlation analysis in micrographs of cells and tissues allows investigators to assess subcellular compartment specific protein complex assembly^6^,^7^. Since PCC is a measure of covariance between two fluorescent signals, it serves as a more reliable tool to quantify the extent to which two proteins are part of a multi-molecular complex than conventional colocalization analysis^8^. In this article we demonstrate Pearson’s Correlation Coefficient (PCC) analysis robustly quantifies many stages of epithelial adherens junction complex assembly/disassembly dynamics following *de novo* cell-cell contact.

We used formation of the E-cadherin mechano-transduction sensor as a model for multi-protein complex assembly in MDCK cells^9^. Using the calcium switch approach^10^ we quantified several aspects of the mechano-transduction apparatus during monolayer assembly: the formation and trafficking of the minimal cadherin-catenin complex, F-actin anchoring of cadherin complexes and, correlation of α-catenin/F-actin interaction to established tissue tension profiles^11^. Finally, we show this quantitative approach based on measuring covariance, accurately assesses adherens junction complex assembly dynamics in live cells using inexpensive image acquisition equipment while minimizing false-positives caused by non-specific signal overlap.

## Results

### Quantifying cadherin mechano-transduction complex assembly/disassembly following *de novo* cell-cell contact using fluorescence covariance

**Figure 1:**
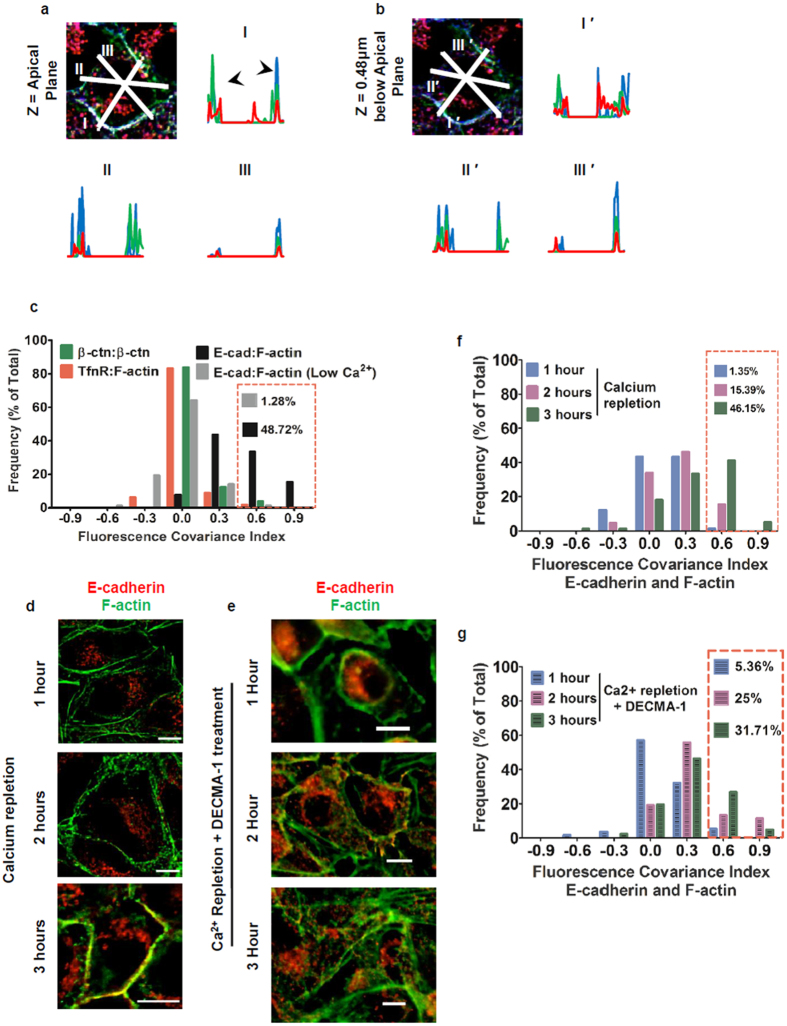
Rational development of *F*luorescence *C*ovariance *I*ndex (FCI) to quantify tissue level protein complex assembly. (**a**) Optical section of MDCK cells 3 hours after calcium repletion immunostained for E-cadherin (red), F-actin (green) and β-catenin (blue). (**b**) Optical section 0.48 μm below section in **a**. Graphs show intensity plots along analysis lines I, II, and III in **a** and **b**. Y-axis represents Intensity (a.u.) and X-axis represents Distance (μm). (**c**) Frequency plots of FCI values for TfR and F-actin (Red bars, n = 113), β-catenin immunostained by two antibodies (Green bars, n = 130), E-cadherin and F-actin at steady state (Black bars, n = 78) and, E-cadherin and F-actin following extra-cellular calcium chelation (Grey bars, n = 78). (**d,e**) Optical sections of MDCK cells 1 hour, 2 hours and 3 hours after calcium repletion (**d**) control or (**e**) with E-cadherin function blocking antibody. Cells were immunostained for E-cadherin (red) and F-actin (green). Scale bars = 10μm. (**f,g**) Frequency plots for FCI values for E-cadherin and F-actin following calcium repletion in (**f**) control condition or (**g**) with E-cadherin function blocking antibody. Bin width = 0.3. After calcium repletion for control: 1 hour (n = 74), 2 hours (n = 65) and 3 hours (n = 78); after calcium repletion with DECMA-1: 1 hour (n = 56), 2 hours (n = 52) and 3 hours (n = 41). Red boxes indicate the percentage of high FCI values (0.5 – 1.0) for each case. Scale bar = 10μm

The cadherin adherens junction mechano-transduction complex functions by coupling tissue tension to cytoskeletal remodeling^12^,^13^. E-cadherin, β-catenin and α-catenin form a minimal cadherin-catenin complex, which directly binds the actin cytoskeleton in response to acto-myosin generated tension^14^. Historically, multi-protein complexes important for epithelial cell-cell adhesion were studied using biochemical assays^15^,^16^. Alternatively, the sub-cellular localization of individual complex components has typically been assessed using immunofluorescence microscopy where complex assembly sites are shown as areas with co-localization of two or more complex component proteins. An early method to assess co-localization was line scan analysis, where the fluorescence intensity of two or more labeled components of the complex along a user defined line is plotted. For instance, line scan analysis in MDCK cells 3-hours following *de novo* cell-cell contact demonstrates E-cadherin, β-catenin and F-actin fluorescence signal overlap at contact sites. This is shown as co-occurrence of fluorescence peaks in the line scan at cell-cell contacts (Fig. 1a). The resulting intensity profiles show overlap in fluorescence peak intensities at the cell-cell contacts indicating the formation of adherens junction complexes at these sites (Fig. 1a, line profile I). However, results of line scan analyses vary significantly depending on the user defined position of the analysis line. Analyzing line scans across different diameters of a cell demonstrate the absence of one or more components of the adherens junction complex along the cell-cell interfaces (Fig. 1a, line profiles II and III). These variations stem from the inherent heterogeneity in the distribution of adherens junction complexes along cell-cell interfaces^17^. Additionally, differences in the distribution of adherens junction complexes along the lateral interface of cells^18^ translate to differences in distribution of adherens junction complexes at different positions along the cell’s z-axis. This is seen as variations in peak fluorescence intensities and overlaps for line scan profiles of analogous lines across multiple optical sections (Fig. 1b). Calculating co-localization or overlap coefficients^3^ using the entire volume occupied by the lateral interface circumvents some of the problems inherent to one dimensional line scans. Given the voxel size is significantly larger than the size of a single cadherin-catenin complex^19^, calculating *M*ander’s *O*verlap *C*oefficients (MOC) (Equation 1) results in erroneously overestimating complex assembly. However, quantifying fluorescence signal covariance using *P*earson’s *C*orrelation *C*oefficients (PCC) (Equation 2)^6^ at cell-cell contacts for E-cadherin and F-actin more accurately measures the extent of their association with the same macromolecular complex.
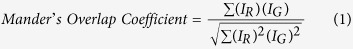

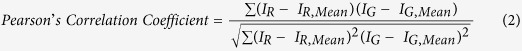


To test this, we calculated MOC and PCC values for three different combinations of molecules: (1) As a negative control, Transferrin receptor (TfR) and F-actin; which have little interaction in any given sub-cellular compartment; (2) As a positive control, β-catenin immunostained by two antibodies with overlapping epitopes; which should have a near complete overlap and covariance in all sub-cellular compartments and; (3) E-cadherin and F-actin; which form a complex at a specific subcellular compartment – the cell-cell interfaces.

MOC and PCC values were calculated for each cell throughout the cytoplasm and at cell-cell contacts (Supplementary Fig. 1a, dotted ROI for cell-cell contact and cytoplasm excluding nucleus). Negative PCC values for TfR and F-actin at cell-cell contacts indicate mutual exclusion of the two signals. This was expected due to the sequestration of a significant population of actin at cell-cell contacts, while TfR is randomly distributed throughout the cell periphery. Importantly, MOC values for TfR showed a small degree of co-occurrence of the two signals demonstrating the degree that non-specific overlap drives “co-localization” of two non-interacting reporter pairs (Supplementary Fig. 1a, TfR:F-actin at cell-cell contacts). However, PCC values for the positive control, β-catenin stained with two antibodies, showed very strong correlation at cell-cell contacts. The MOC values were also high for the two fluorescence signals as would be expected for signals from overlapping epitopes (Supplementary Fig. 1a, β-catenin:β-catenin at cell-cell contacts). E-cadherin and F-actin, which are both part of the adherens junction at cell-cell contacts showed strong correlation values similar to the positive control. MOC values for E-cadherin and F-actin indicate a great degree of overlap of the two molecules at the cell-cell contacts. However, while the MOC values only indicate the degree of overlap, the PCC values indicate the nature of association. For instance, F-actin and TfR are mutually excluded at cell-cell contacts, while E-cadherin and F-actin are strongly associated with each other at cell-cell contacts (Supplementary Fig. 1a). In the cytoplasmic compartment, where TfR and F-actin are both randomly distributed, PCC values show a “very weak” correlation and MOC values indicate weak overlap of the two signals. In contrast, β-catenin signals localized to the perinuclear ER-golgi like distribution show strong correlation and overlap, as expected from overlapping epitopes. Importantly, the PCC and MOC values for E-cadherin and F-actin in the cytoplasm are low, resembling randomly distributed signals of TfR and F-actin in this compartment (Supplementary Fig. 1a, cytoplasm). These results demonstrate the proof of principle that using PCC values reveals the nature of association between two molecules, whether they are strongly associated, very weakly associated or mutually excluded.

In order to study the behavior of the cell populations as opposed to single cells, frequency plots of PCC values for the above three combinations of molecules in multiple cells were graphed (Materials and Methods). The distribution for E-cadherin and F-actin in the cytoplasmic compartment was centered at 0, resembling that of TfR and F-actin. However, the frequency plot for the two β-catenin signals was significantly biased toward higher correlations (Supplementary Fig. 2a). Since the two β-catenin signals in the perinuclear compartment recognize the same molecule, the high correlations are expected. In contrast, frequency plot of PCC values for TfR and F-actin at cell-cell contacts showed a distribution centered at 0, while the frequency plot for E-cadherin and F-actin was biased towards high correlations resembling that of the two β-catenin signals (Supplementary Fig. 2b). These results demonstrate a substantial proportion of cells have E-cadherin and F-actin in a complex at cell-cell contacts, while the two molecules have very little interaction in the cytoplasm.

To obtain a single metric quantifying the extent of adherens junction maturation taking into account cell-cell contact and cytoplasmic correlations (or lamellipodial overlap containing lateral/*en face* adhesions; for a more detailed explanation of *en face* adhesions see section on α-catenin and F-actin below), the ratio of PCC values at cell-cell contacts to PCC values in the cytoplasm for E-cadherin and F-actin was logarithm transformed (Equation 3). This measure, termed *F*luorescence *C*ovariance *I*ndex (FCI) represents the relative asymmetry in correlation between the two compartments: cell-cell contacts and cytoplasm.
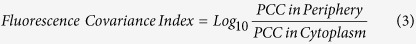


FCI values given by equation 3 have no bounds and are susceptible to large variations depending on the relative PCC values between the two compartments being analyzed. Since, FCI measures the asymmetry in correlations between the two compartments, PCC values lower than 0.1 were reassigned to 0.1, a value representing “very weak” correlation^20^. Thresholding PCC values constrains FCI values to the range: −1 to +1 and eliminates undefined values due to negative PCCs. Importantly, PCC values between 0.1 and 0 carry little biological significance justifying the thresholds applied to measure the relative asymmetry in covariance^6^,^20^. FCI values between −1 and 0 signify higher PCCs in the cytoplasm, while values between 0 and +1 signify higher PCCs at cell-cell contacts. Thus, FCI values indicate the relative contribution of positive correlations at cell-cell contacts and in the cytoplasm, providing a single number to assess the extent of apical and lateral/*en face* adherens junctions.

To test the effects of setting a threshold on PCC values, frequency distributions of PCC values in multiple cells were re-plotted after setting thresholds for the three combinations of molecules: TfR and F-actin, β-catenin stained with two antibodies with overlapping epitopes and, E-cadherin and F-actin. The frequency plots for PCC values in both cellular compartments for the two β-catenin signals and, E-cadherin and F-actin remained largely unaffected after setting the threshold. However, the distribution for TfR and F-actin show a significant right shift since the two molecules were mutually excluded (large negative PCC values) in several cells (Supplementary Fig. 2c,d).

The frequency distributions of FCI values for the three combinations of molecules were plotted to determine which combination showed a relative asymmetry in covariance in the two sub-cellular compartments. TfR and F-actin showed very weak covariance in both compartments and, the two β-catenin signals were highly correlated in both compartments (Fig. 1c, green and red bars). The FCI distributions for these two combinations are centered at 0 representing a “null hypothesis” or no asymmetry in covariance between the two sub-cellular compartments. In contrast, E-cadherin and F-actin showed a right shifted distribution, indicating a cell-cell contact biased asymmetry in E-cadherin and F-actin association owing to adherens junction maturation (Fig. 1c, black bars). Additionally, upon chelation of extracellular calcium, E-cadherin and F-actin FCI distribution shifted to the left, resembling the null hypothesis distribution. This stems from a disassembly of adherens junction complex upon chelation of extra-cellular calcium, resulting in a loss of asymmetry in correlation between the two molecules in either sub-cellular compartment. While steady state cells had ≈49% FCI values ranging between 0.5 and 1.0, only ≈1% of FCI values fell within this range upon calcium chelation (Fig. 1c, grey bars). These results establish the derivation of FCI values and, the dynamic range of the metric to assess adherens junction assembly: adherens junction maturation is represented if the frequency of cells with FCI values for E-cadherin and F-actin in 0.5–1.0 range is ≥45%; and adherens junction disassembly is indicated by a frequency distribution biased towards 0.0–0.5 range.

In order to carry out a quantitative assessment of fluorescence covariance across multiple optical sections and include the entire 3-dimensional volume of the cell, Z-stacks were acquired using a high Quantum Efficiency CCD camera. Out-of-focus contributions due to widefield acquisition were reassigned and images were restored using the Constrained Iterative Maximum Likelihood Algorithm for deconvolution (Supplementary Fig. 3)^1^,^21^. Since a change in absolute fluorescence intensity of either one or both of the signals does not affect PCCs, a linear normalization function was applied (See Materials and Methods). However, noisy images show high PCC values due to an inability to accurately converge on a solution by the image restoration algorithm^4^. Therefore, images with a low Signal-to-Noise Ratio (SNR) cause higher cytoplasmic PCCs arising from signal (or more accurately, noise) overlap rather than lateral/*en face* adhesions and, ultimately underestimating the extent of complex assembly at cell-cell contacts. Exposure times were reduced up to ten fold to obtain images with low SNR and the resultant FCIs for E-cadherin and F-actin were measured (Supplementary Fig. 4a). Lowering the exposure times reduced the proportion of cells with high (0.5–1.0) FCI values (Supplementary Fig. 4b). Only a 10 fold reduction in exposure time showed a substantial drop in FCI values (≈37% in 0.5–1.0 range for 10 fold reduction in intensity and >45% for all other exposure times), indicating FCI measurements can tolerate small changes in signal intensity but are sensitive to very noisy (high noise) images (Supplementary Fig. 4c).

Another image processing parameter which could potentially effect FCI measurements is the degree of image restoration by deconvolution. This was tested by varying the deconvolution “strength” parameter, thereby increasing stringency and decreasing out-of-focus contribution in the original image^22^. Low stringency deconvolution generates images with significant out-of-focus contribution due to poor image restoration (Supplementary Fig. 4d). This resulted in a lower proportion of cells with high FCI values. Better image restoration correlates with an increase in the frequency of high FCI values (Supplementary Fig. 4e, ≈34% in 0.5–1.0 range for high image noise and >45% for all other strength settings). However, there was no significant change in FCI values when images were restored with medium, high or very high stringency (Supplementary Fig. 4f). These data demonstrate FCI values exhibit minimal variation for a wide range of image restoration parameters.

Having optimized the image acquisition criteria and validated the FCI analysis as a measure of asymmetry in correlation between two compartments, and hence a quantitation of protein complex assembly, the method was used to assess adherens junction assembly. Synchronized *de novo* cell-cell contacts were initiated using a calcium repletion assay^10^. There was a progressive increase in E-cadherin fluorescence intensity and co-localization with F-actin fluorescence intensity at cell-cell contacts as adherens junctions are assembled (Fig. 1d). Correspondingly, frequency distribution of PCC values showed an increasing proportion of cells with higher PCCs for E-cadherin and F-actin at cell-cell contacts from 1 through 3 hours after calcium repletion indicating adherens junction assembly (Supplementary Fig. 5a). Conversely, PCC values for E-cadherin and F-actin in the cytoplasm were low following calcium repletion (Supplementary Fig. 5b), demonstrating little E-cadherin and F-actin association in the cytoplasm. To further validate the extent to which PCC values for E-cadherin and F-actin reflect adherens junction assembly, E-cadherin homophilic binding was inhibited using E-cadherin function blocking antibodies during calcium repletion^23^. After blocking E-cadherin function, the cells fail to progressively accumulate E-cadherin at the cell-cell contacts and showed significant intercellular overlap following calcium repletion (Fig. 1e). Intriguingly after inactivating E-cadherin function with function blocking antibodies, E-cadherin and F-actin fluorescence covaries demonstrated by increased PCC values at cell-cell contact sites between 1 and 2 hours after calcium repletion. However, 3 hours after calcium repletion with E-cadherin function blocking antibody, cells failed to undergo adherens junction maturation evidenced by the frequency distribution of PCC values shifting significantly to the left (Supplementary Fig. 5c). Additionally, significant lamellipod overlap following calcium repletion caused high PCC values for E-cadherin and F-actin in the cytoplasm (Supplementary Fig. 5d).

Consistent with adherens junction assembly in control conditions, there is a progressive increase in the proportion of cells with high FCI values following calcium repletion over a period of 3 hours (Fig. 1f). 3 hours after calcium repletion, the proportion of cells with high FCI values is comparable to that at steady state (≈46% at 3 hours following calcium repletion compared to ≈49% at steady state), indicating *de novo* adherens junction assembly and maturation occurs within this time frame as supported by previous reports^24^ (Fig. 1f). Finally, frequency distribution plots of FCI values for E-cadherin and F-actin during the entire course of a calcium repletion experiment were fit to Gaussian distributions and show a progressive increase in mean FCI values. The mean of the 3 hour distribution overlaps with the steady state value following calcium repletion (Supplementary Fig. 6a). Additionally, FCI mean values at 3 hours were not significantly different from those at steady state, confirming adherens junction maturation by 3 hours after *de novo* cell-cell contact (Supplementary Fig. 6b). However, cells treated with E-cadherin function blocking antibody fail to reach the steady state proportion of high FCI values for E-cadherin and F-actin (≈31% at 3 hours following calcium repletion with E-cadherin function blocking antibody compared to ≈49% at steady state), indicating an inability to fully assemble mature adherens junctions (Fig. 1g). These results demonstrate FCI analysis for E-cadherin and F-actin is sensitive to E-cadherin function, and the frequency of high FCI values provides a quantitative measure of adherens junction assembly/disassembly dynamics in epithelial monolayers.

Finally, to test whether the PCC analysis can be used to measure the extent to which a protein exists in different conformational states in a macromolecular complex, we chose to determine the activation state of E-cadherin during adherens junction assembly. E-cadherin exists in several conformational states, whose abundance depends on several factors including the presence of extracellular calcium and, the phosphorylation state of p120-catenin. These isoforms can be distinguished by different antibodies^25^. DECMA-1 is a rat monoclonal E-cadherin function blocking antibody that binds to an extracellular domain of the protein in the presence of calcium, while the mouse monoclonal antibody from BD Biosciences (clone 36) detects adhesion activated E-cadherin^26^. Activation of E-cadherin to an adhesion competent conformation exposes the cytoplasmic epitope for the clone 36 mouse monoclonal antibody^25^. In order to determine the extent of adhesion activated E-cadherin at cell-cell contacts during adherens junction assembly and maturation, E-cadherin was double labeled with DECMA-1 and clone 36 antibodies during a calcium repletion experiment. Cells in steady state showed a significant proportion of E-cadherin present at the cell-cell contacts stained by DECMA-1 was in fact adhesion activated and, was co-stained by clone 36 antibody (Supplementary Fig. 7a, steady state). However, during adherens junction formation following calcium repletion, DECMA-1 staining localized to cell-cell contacts to a greater extent than clone 36 antibody staining (Supplementary Fig. 7a, calcium repletion), Additionally, a progressive increase in clone 36 antibody staining at cell-cell contacts was observed during calcium repletion, indicating E-cadherin becomes adhesion activated during the assembly of adherens junctions. In order to determine the extent of covariance between F-actin and E-cadherin stained with either DECMA-1 or clone 36, cells were co-labelled with fluorescently tagged phalloidin. A map of the positive Product of Differences from Mean (+PDM) showed higher correlations in the cell-cell contact zone between DECMA-1 and F-actin compared to clone 36 and F-actin (Supplementary Fig. 7a′ arrows). Correspondingly, PCCs at the cell-cell contact sites between clone 36 and F-actin and, DECMA-1 and F-actin showed a small but significant variation at all time points during calcium repletion (Supplementary Fig. 7b). Since clone 36 only recognizes adhesion activated E-cadherin, which is a subset of the total E-cadherin present at cell-cell contacts, the PCC values between clone 36 and F-actin were lower than those between DECMA-1 and F-actin. Adhesion competent (or activated) E-cadherin is required to anchor functional adherens junction complexes to F-actin. Hence, clone 36 antibody was used to assess the covariance of E-cadherin and F-actin and the corresponding FCI values (Fig. 1 and Supplementary Figs 1–6) were used as a measure of adherens junction assembly/disassembly.

### Fluorescence covariance analysis quantifies minimal cadherin-catenin complex assembly in the perinuclear cytoplasm and subsequent accumulation at cell-cell contacts following *de novo* contact

**Figure 2:**
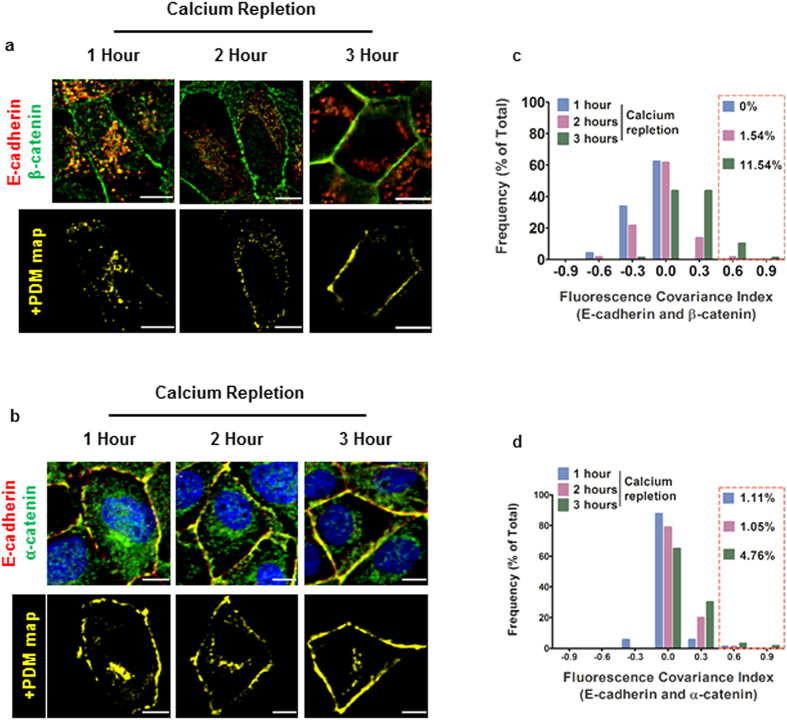
Formation of “minimal” cadherin-catenin complex in the perinuclear cytoplasm and subsequent localization to cell-cell contacts. (**a**) **Top panel**: MDCK cells in 1 hour, 2 hours and 3 hours after calcium repletion immunostained for E-cadherin (red) and β-catenin (green); **Bottom panel**: +PDM maps for the images shown in the top panel. (**b**) **Top panel**: MDCK cells in 1 hour, 2 hours and 3 hours after calcium repletion immunostained for E-cadherin (red) and α-catenin (green); **Bottom panel**: +PDM maps for the images shown in the top panel. (**c**) Frequency plots of FCI values for E-cadherin and β-catenin (with bin width = 0.3) at 1 hour (n = 74), 2 hours (n = 65) and 3 hours (n = 78) after calcium repletion. (**d**) Frequency plots of FCI values for E-cadherin and α-catenin (with bin width = 0.3) at 1 hour (n = 90), 2 hours (n = 95) and 3 hours (n = 63 cells) after calcium repletion. Scale bar = 10μm and red boxes indicate the percentage of high FCI values (0.5–1.0) for each case.

E-cadherin binds β-catenin, which in turn binds α-catenin in the ER-Golgi compartments prior to its trafficking to the apico-lateral surface of contacting epithelial cells^16^,^27^,^28^. A significant proportion of E-cadherin localizes with β-catenin and α-catenin in the cytoplasm and correspondingly, positive correlations were observed between E-cadherin and β-catenin and, E-cadherin and α-catenin, in the cytoplasm at 1 and 2 hours following calcium repletion (Fig. 2a,b: 1 and 2 hours and, Supplementary Fig. 8a,b). Conversely, increasing co-localization between E-cadherin and β-catenin and, E-cadherin and α-catenin is observed along with increasing positive correlations for the two combinations of molecules at cell-cell contacts from 1 through 3 hours after calcium repletion (Fig. 2a,b and Supplementary Fig. 8c,d). Also, positive cytoplasmic correlations progressively reduced, which together with the increasing correlations at cell-cell contacts indicate trafficking of the minimal cadherin-catenin complex from the perinuclear biosynthetic compartment to sites of adherens junction assembly. Frequency distribution plots of FCI values for the cadherin-catenin complexes show a progressive increase in the accumulation of cadherin-catenin complexes at the cell-cell contacts (Fig. 2c,d). These data indicate FCI values can be used to track the relative contribution of preassembled minimal cadherin-catenin complexes in the perinuclear cytoplasm to those at cell-cell contacts.

Microtubules are known to interact with adherens junction complexes with a geometry perpendicular to the actin filaments at these intercellular junctions^29^,^30^,^31^,^32^,^33^. The microtubule network at cell-cell contacts is significantly less dense than the cortical F-actin network (Supplementary Fig. 9a, α-tubulin staining in steady state, compare with Supplementary Fig. 1a, F-actin staining). This suggests the extent of microtubule and adherens junction correlation should be significantly lower than F-actin and adherens junction. As per the prediction, MDCK cells in steady state showed some positive correlations between β-catenin and α-tubulin at cell-cell contacts. The extent of this correlation was much lower than those observed between E-cadherin and F-actin (Supplementary Fig. 9a compare +PDM map at steady state to Supplementary Fig. 1a +PDM map for E-cadherin and F-actin). Interestingly, 1 hour following calcium repletion, punctate positive correlations for β-catenin and α-tubulin were observed in the cytoplasm (Supplementary Fig. 9a, 1 hour, arrowheads). These cytoplasmic correlations decreased 2 and 3-hours after calcium repletion (Supplementary Fig. 9a,b). Given cadherin-catenin complexes are assembled in the perinuclear cytoplasm following translation^16^,^27^, the positive correlation of β-catenin with α-tubulin in the cytoplasm 1 hour following calcium repletion most likely represents cadherin-catenin complexes being trafficked along microtubules. Most cells showed little positive correlation between α-tubulin and β-catenin at cell-cell contact sites through the course of calcium repletion for 3 hours (Supplementary Fig. 9c). Some punctate positive correlations were observed at a few cell-cell contacts 3 hours after calcium repletion (Supplementary Fig. 9a, 3 hour), but the overall correlation coefficients were low. Lastly, frequency distribution plots of FCI values for β-catenin and α-tubulin fit normal distributions centered at zero for low calcium, 1, 2 and 3-hours after calcium repletion. A small positive mean FCI value for steady state distribution demonstrates microtubules associate with adherens junctions, albeit on a slower time scale and to a lesser extent than actin filaments (Supplementary Fig. 9d). The following four conclusions can be drawn from these results. First, microtubule and adherens junction interactions require longer time scales than adherens junctions anchoring to actin. Second, the extent of microtubule and adherens junction interaction is lower than F-actin and adherens junction interaction at cell-cell contacts. Third, the minimal cadherin-catenin complex assembles in the perinuclear cytoplasm and, likely traffics on microtubules before reaching the cell-cell contact. Lastly, given the frequency distributions of FCI values for α-tubulin and β-catenin are all almost perfectly centered at zero, the right shift seen in the frequencies of FCI values for the cadherin-catenin complexes represents a biologically significant accumulation of these complexes at the cell-cell contacts, in spite of the very low frequency of high FCI values (Fig. 2).

### FCI analysis of α-catenin and F-actin correlates with established tissue tension profile during assembly and maturation of adherens junctions

**Figure 3:**
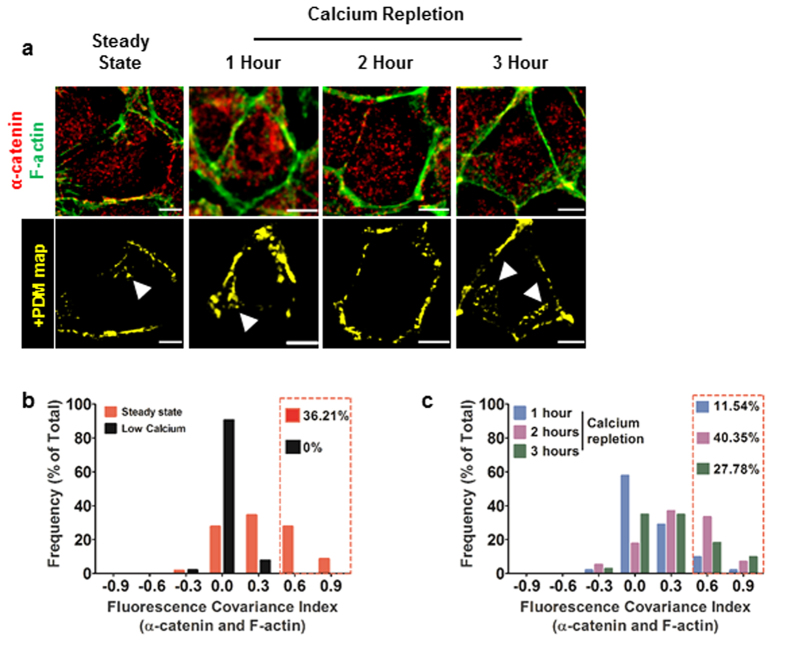
α-catenin and F-actin interactions correlate with established tissue tension profiles. (**a**) **Top panel:** MDCK cells in steady state, 1 hour, 2 hours and 3 hours after calcium repletion immunostained for F-actin (green) and α-catenin (red). Scale bar = 10 μm. **Bottom panel:** +PDM maps for the images shown in the top panel. (**b**) Frequency plots for FCI measurements at steady state (n = 58) and low calcium (n = 52). (**c**) Frequency plots for FCI measurements at 1 hour (n = 52), 2 hours (n = 57) and 3 hours (n = 72) after calcium repletion. Red boxes indicate the percentage of medium and high FCI values (0.5–1.0) for each case and bin width = 0.3.

α-catenin is a tension sensitive component of two intercellular adhesion systems: adherens junctions and tight junctions^14^,^30^,^34^,^35^,^36^. In a polarized epithelial tissue, adherens junctions are subject to a circumferential myosin IIB dependent tension restricted to the apical region^36^ and myosin IIA dependent tension along the basolateral membrane^18^. As a result, the basolateral plasma membranes develop protrusions which contain what appear as lateral/*en face* adherens junctions^37^,^38^. On the other hand, during the initial phases of adherens junction assembly, cells form lamellar overlaps which contain adherens junction components. These nascent adhesion complexes are then trafficked in a basal-to-apical manner as cells lift and polarize^39^,^40^. Atomic force microscopic measurements in an assembling epithelial monolayer have shown that myosin II dependent tension peaks between 2 and 3 hours following calcium repletion^11^. The two tension profiles, myosin IIB dependent apical and myosin IIA dependent lateral tension components balance each other resulting in the formation of a polarized columnar epithelium. In view of these findings and evidence that α-catenin binding to F-actin is tension sensitive, cells were co-stained for α-catenin and F-actin and FCI analysis was carried out to determine the dynamics of interactions between these two molecules during *de novo* adherens junction assembly. Positive correlations in the cytoplasm were observed at steady state, 1 and 3 hours after calcium repletion. These appear as contributions from lateral adhesions and lamellar overlaps (Fig. 3a arrowheads in +PDM maps). However, 2 hours after calcium repletion cells showed significantly less cytoplasmic correlations (Fig. 3a, 2 hour). Additionally, calcium chelation resulted in loss of high frequency FCI values (≈36% at steady state compared to 0% in low calcium) indicating disassembly of adherens junction complexes results in decoupling of α-catenin and F-actin interaction (Fig. 3b). The proportion of cells with high FCI values for α-catenin and F-actin is greatest at 2 hours following calcium repletion (Fig. 3c, ≈40% at 2 hours compared to ≈12% and ≈28% at 1 and 3 hours, respectively). This increase is due to a corresponding decrease (left shift in frequency plot) in cytoplasmic PCC values (Supplementary Fig. 10a). However, PCC values at cell-cell contacts during steady state and 3 hours after calcium repletion are slightly right shifted compared to 2 hours after calcium repletion (Supplementary Fig. 10b), this is due to the contribution from lateral adhesions appearing in the cytoplasmic compartment following polarization decreased the corresponding FCI values (Supplementary Fig. 10c). These data demonstrate FCI values of α-catenin and F-actin correlate with established tension profiles during the assembly and maturation of adherens junctions (Supplementary Fig. 10d).

### Live cell FCI analysis tracks adherens junction assembly/disassembly dynamics with a 5-minute temporal resolution

**Figure 4:**
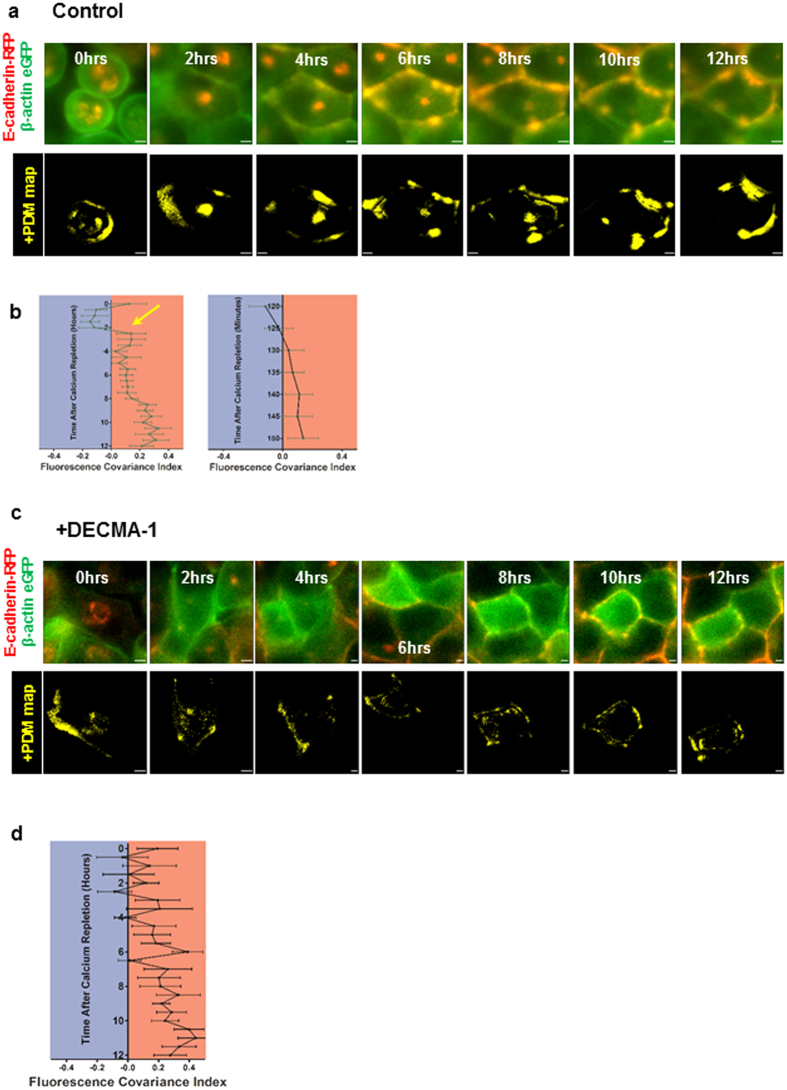
Live cell FCI analysis tracks protein complex assembly using fluorescent fusion tags with 5 minute temporal resolution. (**a**) Top panel: Montage of live cell movie (0 hour–12 hours) showing MDCK cells expressing E-cadherin-RFP (red) and β-actin-eGFP (green) after calcium repletion. Bottom panel: +PDM maps of images from the top panel. (**b**) Left: FCI plotted every 30 minutes for E-cadherin-RFP and β-actin-eGFP after calcium repletion (n = 9). Arrow indicates the time at which FCI attains positive values. Right: FCI plotted every 5 minutes for E-cadherin-RFP and β-actin-eGFP between 2 and 2.5 hours after calcium repletion. Error bars indicate mean ± s.e.m. (**c**) Top panel: Montage of live cell movie (0 hour–12 hours) showing MDCK cells expressing E-cadherin-RFP (red) and β-actin-eGFP (green) treated with DECMA-1 antibody after calcium repletion. Bottom panel: +PDM maps of images from the top panel. (**d**) FCI values plotted every 30 minutes for E-cadherin-RFP and β-actin-eGFP after calcium repletion (n = 6). Arrow indicates the time at which FCI is positive and remains positive through the rest of the experiment. Error bars indicate mean ± s.e.m.

Expressing fluorescently tagged proteins allows high temporal resolution analysis of single cells within a living tissue with high temporal resolution. Using MDCK cells expressing E-cadherin-RFP and β-actin-eGFP, adherens junction assembly was tracked in live epithelial monolayers during calcium repletion experiments. β-actin was chosen since it is required for adherens junction assembly and is enriched at the cell-cell contact zone^41^. The molecular mechanism of actin enrichment at cell-cell contacts involves localized β-actin translation^24^. This synthesis event provides sufficient local concentration of monomers to drive barbed end filament polymerization to assemble linear actin filaments. These filaments in turn are required to anchor nascent adherens junction complexes^24^,^42^. Immediately following calcium repletion, three optical sections were acquired using a widefield microscope with an inter-plane distance of 3μm. Nearest neighbor de-blurring was used to reduce out of focus light in each optical section. The number of optical planes was limited by acquisition speed and phototoxicity. Five minute intervals between successive acquisitions provided good temporal resolution without any evidence of phototoxicity. Consistent with the data obtained using fixed cells, 2 to 3-hours after calcium repletion, +PDM maps showed positive correlations between E-cadherin and β-actin at cell-cell contact zones (Fig. 4a). Interestingly, positive correlations for E-cadherin and β-actin were observed in the cytoplasm for the first 6-hours after calcium repletion. These cytoplasmic correlations likely arise from the non-filamentous globular actin (G-actin) and perinuclear E-cadherin, both present in the perinuclear cytoplasm. This G-actin population is thought to function as a storage compartment for monomers, which are incorporated into spatially localized dynamic filament populations^43^. Plotting FCI values as a function of time showed an inflection point between 2 and 3-hours after calcium repletion (Fig. 4b yellow arrow). This shift from negative to positive FCI values coincided with the appearance of cell-cell contact zone localized positive correlations consistent with the spatio-temporal dynamics of adherens junction assembly (Fig. 4a). During the transition from negative to positive FCI values, measurements were made every 5 minutes to determine adherens junction assembly kinetics with the highest possible temporal resolution for the experimental settings used. A smooth increase in positive correlation in the +PDM maps at cell-cell contact zones was observed during the course of 2 to 2.5 hours following calcium repletion (Fig. 4b′ and Supplementary Fig. 11a). Importantly, the smooth increase in positive correlations was perturbed and delayed by adding E-cadherin function blocking antibody during calcium repletion (Fig. 4c). Upon addition of E-cadherin function blocking antibodies, FCI values showed a fluctuating trend for the first 4.5 hours after calcium repletion. This is caused by cycles of adherens junction assembly and disassembly without any significant anchoring and maturation (Fig. 4d). Additionally, PCC values at cell-cell contact zone and the cytoplasm were substantially reduced for the first 4.5 hours after calcium repletion with E-cadherin function blocking antibody (Supplementary Fig. 11b,c). These results demonstrate live cell FCI analysis quantitates the dynamics of protein-protein interaction within 2 different subcellular compartments with high spatial and temporal resolution.

## Discussion

**Figure 5:**
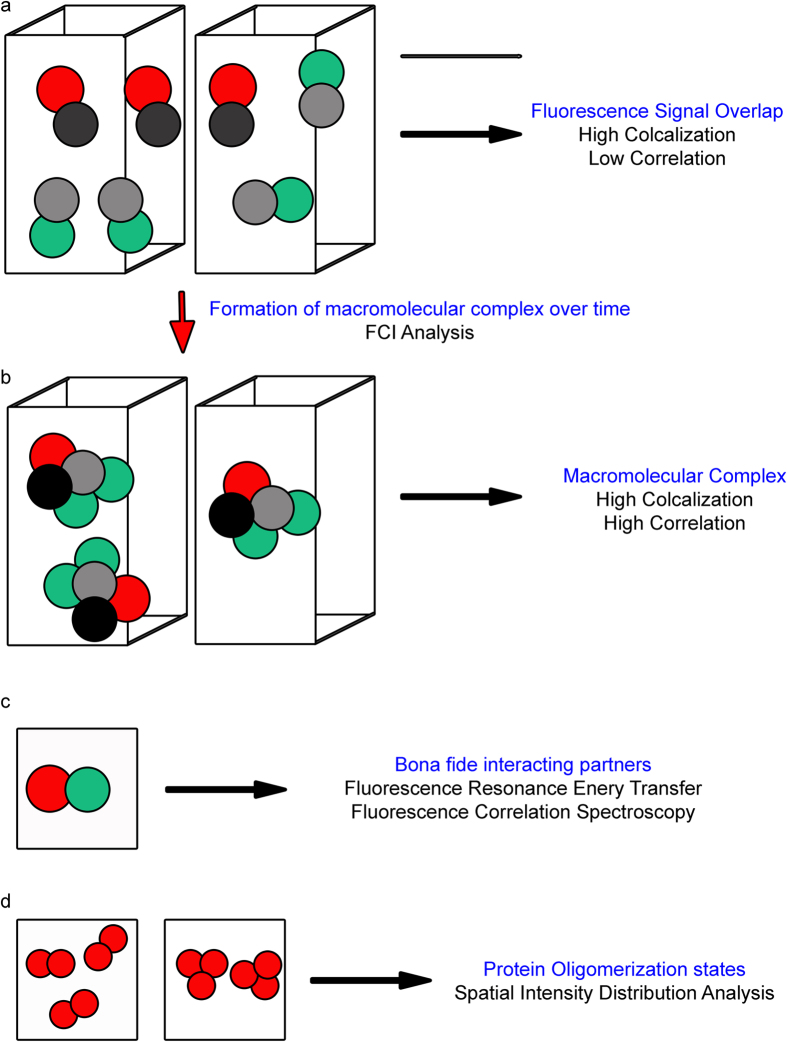
Measuring spatial and temporal dynamics of protein-protein interactions. **(a)** Co-localization of two proteins (Red and Green spheres with two individual voxels. The two proteins do not interact or form a macromolecular complex and are simply present in the same voxel. Although the co-localization coefficients will be high in this case, the correlation will be low since the two proteins are not proportional to one another. **(b)** Macromolecular complex composed of the two proteins being studied (Red and Green spheres). The complex could arise from the situation in **a** over time or in another spatial domain or both. In this situation the co-localization and correlation coefficients will be high. FCI analysis tracks the change from situation in **a** to the situation in **b**, thus providing information about the extent of complex assembly in both time and space. (**c**) Directly interacting proteins at a distance of 5–10 nm can be studied using FRET and FCS studies. (**d**) Protein oligomerization states such as those of transmembrane receptors can be studied by using the spatial domain of intensity distributions or SpIDA.

Analysis of subcellular co-localization to study the formation of protein complexes and protein-protein interactions is riddled with caveats and footnotes^1^,^4^,^6^,^20^. In this article, a method based on Pearson’s Correlation Coefficients is used to demonstrate various aspects of multi-protein complex assembly dynamics pertaining to the assembly of the adherens junction complex in epithelial cells. While, E-cadherin clustering and dynamics at adherens junctions have been studied using super-resolution light microscopy and FRAP analysis^38^,^44^, there have been few analysis tools available to robustly study the spatial and temporal dynamics of macromolecular complexes such as adherens junctions (Fig. 5). To date, the only available methods to study multi-protein complexes are those in a biochemist’s toolbox: immunoprecipitation, sub-cellular fractionation, protein cross-linking and pull down assays. While the utility of these biochemical approaches is not discounted, they are not without their own technical caveats. Alternatively, Spatial Intensity Distribution Analysis (SpIDA)^45^ can be utilized to assess clustering of transmembrane receptors such as E-cadherin during adherens junction assembly. SpIDA analysis is however limited to determining oligomerization states of various components in multiprotein complexes such as adherens junctions (Fig. 5d) (E-cadherin and α-catenin for example)^46^,^47^. However, the goal of the PCC based method presented here is to determine the spatio-temporal dynamics of association of various components of a multi-protein complex during assembly. PCC measurements offer a simple yet robust method to carry out “biochemical analysis on a microscope”, thus having the advantage of being able to provide quantitative spatio-temporal data regarding macromolecular complexes. Fluorescence Covariance Index provides a quantitation of the asymmetry in correlation between two signals within two user defined sub-cellular compartments over time (Fig. 5a,b). This novel method helps one to follow the association of two components in the spatial and temporal domains, thus providing information about where and when a macromolecular complex assembles in the cell. The assumption is that the multi-protein complex which includes the two molecular components being studied forms preferentially in one of the sub-cellular compartments. While defining these two distinct sub-cellular compartments, one needs to bear in mind that the compartment with no protein complex assembly should have a very weak correlation. For instance, FCI analysis of β-catenin and F-actin during a calcium repletion assay, using the cytoplasm and cell-cell contact as the two compartments in the analysis violates this assumption. This is due to the fact that during later phases of adherens junction assembly, β-catenin is sequestered at the cell-cell contact zone by E-cadherin. F-actin is also predominantly present at the cell-cell interface in a polarized epithelium. As a result both signals in the cytoplasm are very low, causing the PCC in cytoplasm to be high (Equation 2). Consequently FCI is very low with a frequency distribution resembling the null hypothesis, where there is no preferred asymmetry in correlation in either compartment (data not shown). However, with the right choice of two proteins and sub-cellular compartments, several behaviors of the adherens junction complex have been quantitated using the FCI analysis. (1) extent of anchoring of the adherens junction complex to the actin cytoskeleton (for example, Fig. 1); (2) tracking the extent of changes in activation of components of the complex over time (for example, Supplementary Fig. 7); (3) tracking minimal cadherin-catenin complexes from biosynthetic compartment to sites of cell-cell adhesions (for example, Fig. 2), (4) correlating the interactions of components of the complex which are tension sensitive to tissue tension profiles (for example, Fig. 3); and (5) correlating the extent of anchoring of the adherens junction to the actin cytoskeleton with tissue barrier function^48^.

The adherens junction complex has a mechano-transduction module: α-catenin and F-actin. Polarized epithelia demonstrate apicolateral and basolateral actomyosin tension profiles mediated by myosin IIB and myosin IIA, respectively^18^,^49^. The balance between these two tension profiles determines whether cells form a functional epithelial tissue^49^ or extrude out of the tissue^50^. Acto-myosin tension in the basolateral compartment results in the appearance of lateral/*en face* adhesions or protrusions, while the apicolateral tension acts to keep the apical zonula adherens intact. FCI analysis is a viable and simple method to study the relative contributions of these two tension components by assessing the interaction of α-catenin and F-actin in these two sub-cellular compartments, using the antibody which recognizes the conformational change in α-catenin subjected to tension^36^.

E-cadherin exists in multiple conformational states^25^,^26^. Adhesion activated E-cadherin is only a subset of E-cadherin present at the cell-cell contacts (Supplementary Fig. 7). E-cadherin function is subject to regulation and can potentially control epithelial tissue fate without any marked changes in its transcriptional state^26^,^51^,^52^, thus a correlation analysis of adhesion activated E-cadherin and its other conformational states could potentially be used as a diagnostic tool to determine the extent of functional E-cadherin mediated adhesions in epithelial cancers. Further, adhesion activated E-cadherin is a scaffold for several signaling molecules including Src family of kinases^53^. It would be interesting to determine whether other conformational states of E-cadherin can function as a scaffold for these signaling cascades. These questions can be tested using the FCI method to analyze the covariance of various E-cadherin conformational states with the appropriate signaling molecules believed to be associated with functional E-cadherin.

This novel technique provides a cost effective, reliable and robust method to assess the extent to which proteins are part of a complex in specific sub-cellular compartments within the resolution limit of a light microscope (Fig. 5). The technique facilitates quantitative comparison of the extent of inhibition/stimulation of protein complex assembly by perturbing different nodes along intracellular signaling pathways. One such example using the formation of the adherens junction complex is: the effect of E-cadherin function on adherens junctions (Figs 1d and 4c). Another novel node in the formation of adherens junctions is localization of β-actin translation to cell-cell contacts^24^,^48^. Such experiments with other multi-protein complexes like the tight junctions^54^, apico-basal polarity determinants^55^, to name a couple, will answer key questions regarding the dynamics of assembly/disassembly of complexes.

Studying protein-protein interactions in living cells is typically carried out using *F*luorescence *R*esonance *E*nergy *T*ransfer (FRET) or *F*luorescence *C*orrelation *S*pectroscopy (FCS)^56^,^57^. However, these techniques require that the two proteins have direct interaction (effective distance for FRET imaging is 5–10 nm) (Fig. 5c). Proteins that do not directly interact, but are part of large multi-protein complexes with several components cannot be studied using either FRET or FCS approaches. Live cell FCI analysis provides a high temporal resolution picture of protein-protein interactions in live tissues without the limitations of FRET experiments (for example, Fig. 4). Using the appropriate fluorescent fusion proteins, this analysis can be easily extended to study for instance, focal adhesion complex assembly dynamics at the leading edge of motile cells, or tight junction assembly in epithelial cells.

## Materials and Methods

### Cell culture

MDCK (Madin-Darby Canine Kidney, ATCC# CCL −34) cells were cultured in DMEM (Corning Cellgro) containing 10% FBS (Life Technologies, Gibco), penicillin (100 I.U./mL) and streptomycin (100μg/mL) (Corning Cellgro). For calcium switch experiments, cells were seeded at a density of 250,000 cells per well in a 6-well plate on glass coverslips (22 × 22 mm) for fixed cell experiments, and 35 mm dishes with glass bottom dishes (MatTek Corp.) for live cell experiments. Cells were grown for 2 d at 37 °C, 95% humidity and 5% CO_2_ to form a monolayer.

### Transfection and generation of stable cell lines

MDCK cells stably expressing E-cadherin-RFP (a gift from Dr. James Nelson, Stanford) were transfected with the plasmid TC-eGFP-β-actin full length (Addgene ID: 27123) using Lipofectamine 2000 (Life Technologies) according to manufacturer guidelines. Cells co-expressing eGFP-β-actin and E-cadherin-RFP were sorted using FACS and used for live-cell experiments. These cells were cultured in regular growth medium along with 500 μg/ml final concentration of G418 to maintain the expression of the reporter genes.

### Calcium switch

To disassemble the adherens junctions in monolayers, the cells were incubated with DMEM (no serum or antibiotics) containing 4 mM EGTA for 1 hour at 37 °C, 95% humidity and 5% CO_2_. Cells were then returned to regular growth media (calcium repletion) and fixed at various time points of the calcium switch experiment (steady state, low calcium, 1, 2 and 3 hours after calcium repletion). For live-cell microscopy, imaging was performed after returning the cells to regular growth media composed of: FluoroBrite DMEM (Life Technologies), FBS (10% v/v), penicillin (100 I.U./mL), streptomycin (100 μg/mL), OxyFluor (1% v/v, Oxyrase) and Sodium dl – Lactate (20 mM, Sigma Aldrich). For fixed cells and live cell imaging E-cadherin function blocking antibody (100 μg/ml, DECMA-1, Sigma Aldrich) was added during calcium repletion.

### Antibodies

The following primary antibodies were used for indirect immunofluorescence staining: mouse anti-E-cadherin (1:250, clone 36, BD Biosciences) was used for immunofluorescence staining in Figs 1, 2a and 3 and, Supplementary Figs 1, 4 and 7, rat anti-E-cadherin (1:250, DECMA-1 from Sigma Aldrich was used for immunofluorescence staining in Fig. 2b and Supplementary Fig. 7, rabbit anti-β-catenin (1:400, Abcam), mouse anti-β-catenin (clone 14, 1:250, BD Biosciences), rabbit anti-Transferrin Receptor (1:100, Abcam), rabbit anti-α-catenin (1:250, BD Sigma), mouse anti-α-tubulin (1:500, Sigma Aldrich). The following secondary antibodies were used for indirect immunofluorescence staining: Alexa Fluor 488 conjugated goat anti-rabbit (1:500, Life Technologies), Cy5 conjugated goat anti-rabbit (1:500, Life Technologies), Cy3 conjugated goat anti-mouse (1:500, Life Technologies), CF640 conjugated goat anti-rat (1:500, Life Technologies). Additionally, for staining F-actin either Alex Fluor 488 or Rhodamine conjugated Phalloidin (1:150, Life Technologies) was used.

### Indirect immunofluorescence

All steps were performed at room temperature unless specified. Cells were fixed with 4% (w/v) paraformaldehyde (Sigma Aldrich) for 20 min and then permeabilized with 0.5% (v/v) Triton (Bio-Rad) for 1 min. 1% (w/v) BSA was used to block for 1 h, following which samples were incubated with primary antibodies overnight at 4 °C. Samples were then incubated with secondary antibodies, Hoechst (1:10,000, GE Healthcare) and Phalloidin for 1.5 h in the dark. Coverslips were mounted onto slides using ProLong Gold (Life Technologies) mounting media and incubated for 24 h at 4 °C in the dark.

### Image acquisition and processing

For indirect immunofluorescence, images were taken on AxioObservor Z1 (Zeiss) using Axiovision 4.8.2 software (Zeiss) equipped with a Plan-Apo 63X oil immersion objective with NA 1.4. 35 z-stacks with a step size of 0.240 μm were acquired for each field. Binning was set to 2 × 2 using a CoolSNAP HQ^2^ (Photometrics). Filter cube sets used were: Blue Channel: 49 (Zeiss), Green Channel: 38HE (Zeiss), Red Channel: Ex:560/40, Em:630/75 and, Far-red Channel: 50 (Zeiss). The following deconvolution parameters were used: theoretical PSF, constrained iterative algorithm, autolinear normalization (See Equation 4 below) and strength setting of medium (or as mentioned in the Supplementary Fig. 4d) for all fixed samples.
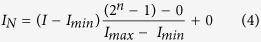
where *I*_*N*_ is the normalized intensity, *I*_*min*_ and *I*_*max*_ are the lowest and highest gray values of the acquired image and *n* is the bit size of the camera used.

For live cell experiments, images were acquired on AxioObservor Z1 (Zeiss), using a 63X water immersion objective with NA 1.3. The stage was equipped with an incubator chamber (37 °C, 95% humidity and 5% CO_2_). QuantEM 512SC camera (Photometrics) was used with no binning. Time-lapse images were taken in 5 minutes intervals for 12 hours, in both RFP (Ex: 560/40 and Em: 630/75) and GFP (Filter Set 38HE, Zeiss) channels. A stack of 3–5 images with a step size of 1 μm was acquired for each time point and nearest neighbor deblurring algorithm was used to process the images. Images were imported to Volocity (Perkin-Elmer) and their brightness adjusted before being exported as a TIF.

### Fluorescence Covariance analyses

Deconvolved images were imported into Volocity 6.0.1 (PerkinElmer) to calculate the PCC values at the cell-cell contact sites and in the cytoplasm. Regions of interest (ROIs) were defined using the Free Hand tool at cell-cell contact and cytoplasm for each cell in an image stack. The PCC values were exported in a comma separated value (.csv) format. A custom written script in MATLAB (MathWorks) was used to extract the PCCs and compute FCI values.

### Statistical Analyses

A parametric ANOVA or non-parametric Kruskal-Wallis test was followed by appropriate post-hoc multiple comparisons tests was performed as indicated in the figure legends. GraphPad Prism 5 (GraphPad Software) was used for all statistical analyses. Frequency distributions were plotted for PCC values and FCI values with bin widths of 0.15 and 0.3 respectively. Bin sizes were determined by using the Sturges’ rule (See Equation 5 below). Gaussian best fits of the frequency distributions for FCI values were obtained with the standard deviation parameter set to be positive.
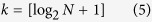
where *k* is number of bins and *N* is sample size. According to this rule, the number of bins will vary depending on sample size. However, it should be noted that while this rule works very well for data with >30 samples, it assumes the population is normally distributed. More importantly, this is still an empirical rule and hence varying bin size could reveal different features of the data. This could become especially important with biologically distinct populations being present which could be falsely grouped into large bins. Conversely, populations that do not arise from different distributions could be split using smaller bins. However, for the sake of the current analysis, the lowest available sample size was about 32, which gives a bin number of 6. This was set as the standard for all frequency plots for FCI values and PCC values (with thresholds).

## Additional Information

**How to cite this article**: Vedula, P. *et al*. Quantifying cadherin mechanotransduction machinery assembly/disassembly dynamics using fluorescence covariance analysis. *Sci. Rep.* **6**, 28822; doi: 10.1038/srep28822 (2016).

## Supplementary Material

Supplementary Information
