# Supplementary material for: Quantifying cadherin mechanotransduction machinery assembly/disassembly dynamics using fluorescence covariance analysis
Source: Sci Rep. 2016 Jun 30;6:28822. doi: 10.1038/srep28822 (PMC4928050; doi:10.1038/srep28822)
Supplement: Supplementary Information [file srep28822-s1.pdf]

Quantifying cadherin mechanotransduction machinery assembly/disassembly dynamics using light microscopy and fluorescence covariance analysis

Pavan Vedula[1, †], Lissette A. Cruz[1, †], Natasha Gutierrez[1], Justin Davis[1], Brian Ayee[1], Rachel Abramczyk[1] & Alexis J. Rodriguez[1, *]

Supplementary Information

Supplementary Figure 1

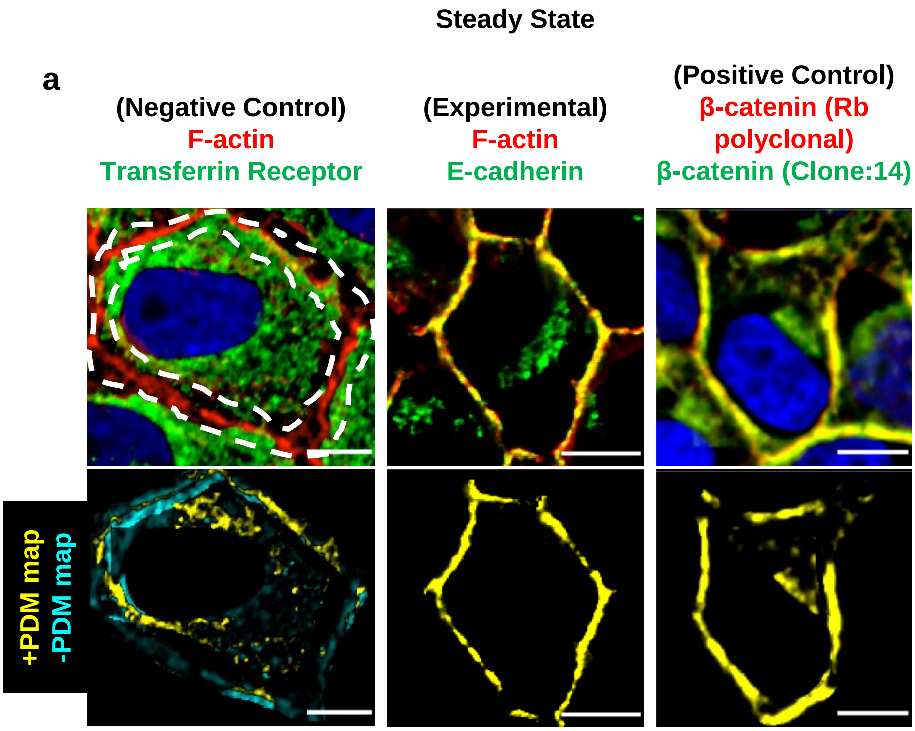

a'

	Transferrin Receptor:F-actin		E-cadherin:F-actin		β-catenin:β-catenin	
	MOC	PCC	MOC	PCC	MOC	PCC
Cell-cell contact	0.288	-0.133	0.903	0.887	0.952	0.921
Cytoplasm	0.398	0.100	0.260	0.237	0.825	0.772

Mutually exclusive and near complete overlap fluorescent signals show FCI values close to zero . (a) Top panel: Images representing MDCK cells in steady state: Left: immunostained for F-actin (Red) and transferrin receptor (Green), Middle: F-actin (Red) and E-cadherin (Green) and Right: immunostained for β-catenin (Rb polyclonal) and β-catenin (Clone:14). **Bottom panel**: +PDM (yellow) and –PDM maps (cyan) for the images shown in top panel. **(a')** Table: calculated Mander's Overlap Coefficients (MOCs) and Pearson's Correlation Coefficients (PCCs) for the cells shown in **(a)**.

Supplementary Figure 2

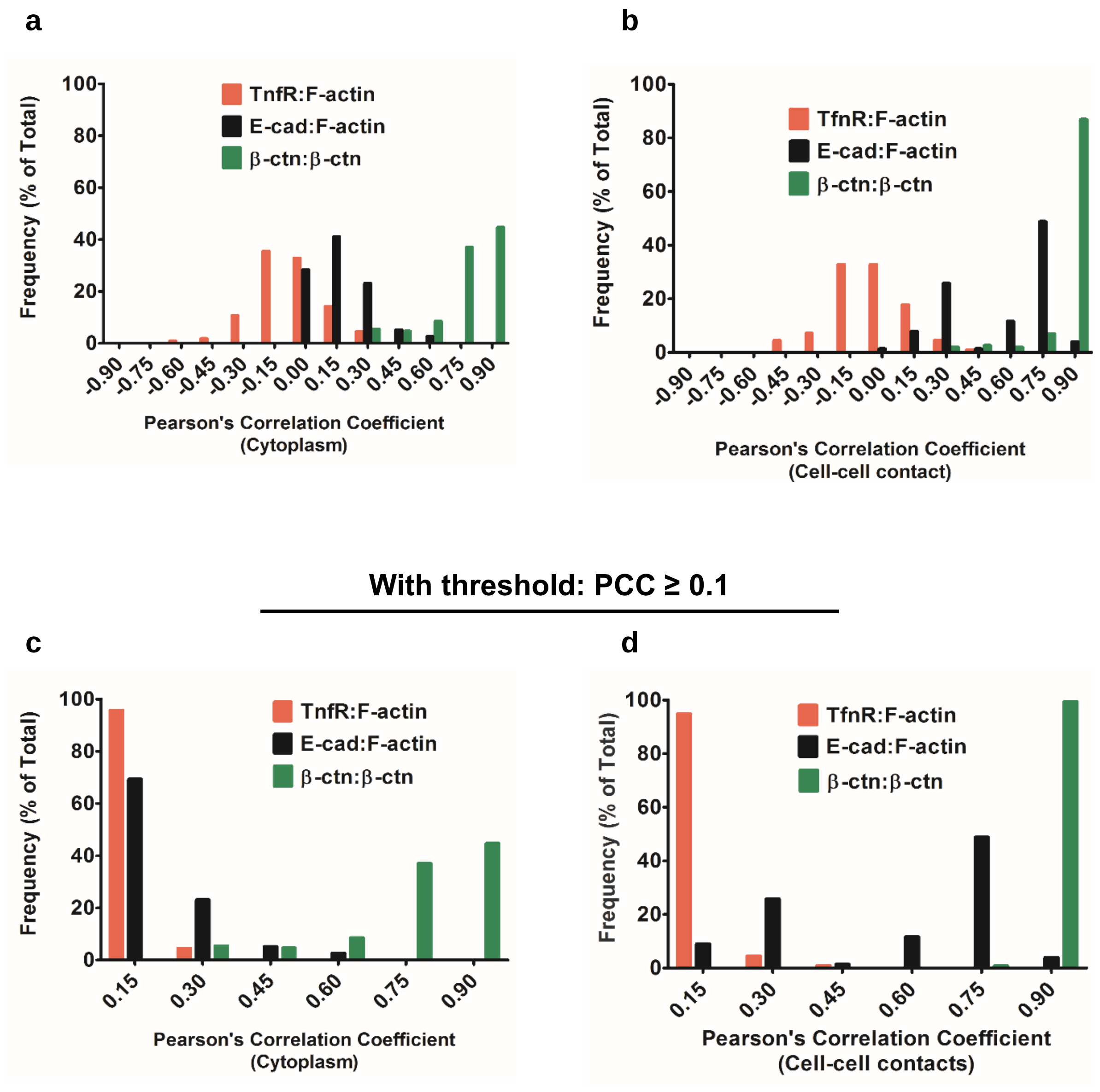

Effects of setting thresholds on PCC values to determine the asymmetry in correlation between cytoplasmic and cell-cel contact compartments. Frequency distributions of (**a, b**) unthresholded and (**c, d**) thresholded PCC values in: cytoplasm (**a, c**) and cell-cell contact zone (**b, d**). Values for: TfR and F-actin are shown as red bars, for β-catenin (Rabbit polyclonal) and β-catenin (Clone:14) are shown as green bars and, for E-cadherin and F-actin are shown as black bars. Bin width = 0.15. The cells were fixed and immunostained in steady state. (**a-d**) N (sample size) values are as follows: Tfr:F-actin = 113, β-catenin:β-catenin = 130, E-cadherin:F-actin in steady state N = 78 and, after calcium chelation for 1 hour N = 78.

Supplementary Figure 3

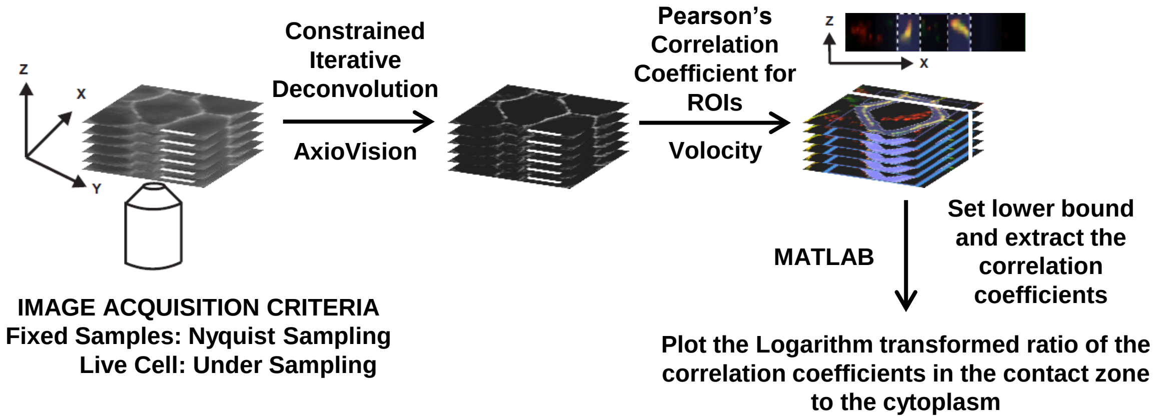

From image acquisition to data analysis: Work flow for FCI analysis. (a) Workflow of image acquisition and processing for measuring FCI. Z-stacks are acquired at nyquist sampling criteria for fixed cells (35 steps of 0.24 μm thickness each to span the entire lateral surface of the cell) and under sampling (3 steps at 3 μm interval between neighboring steps to reduce phototoxicity). Images were deconvolved using constrained iterative algorithm from AxioVision 4.8.2 (Carl Zeiss Microscopy). PCC was computed for defined ROIs – contact zone and cytoplasm – using Volocity 6.0 (Perkin Elmer). FCI values were extracted using MATLAB and plotted.

Supplementary Figure 4

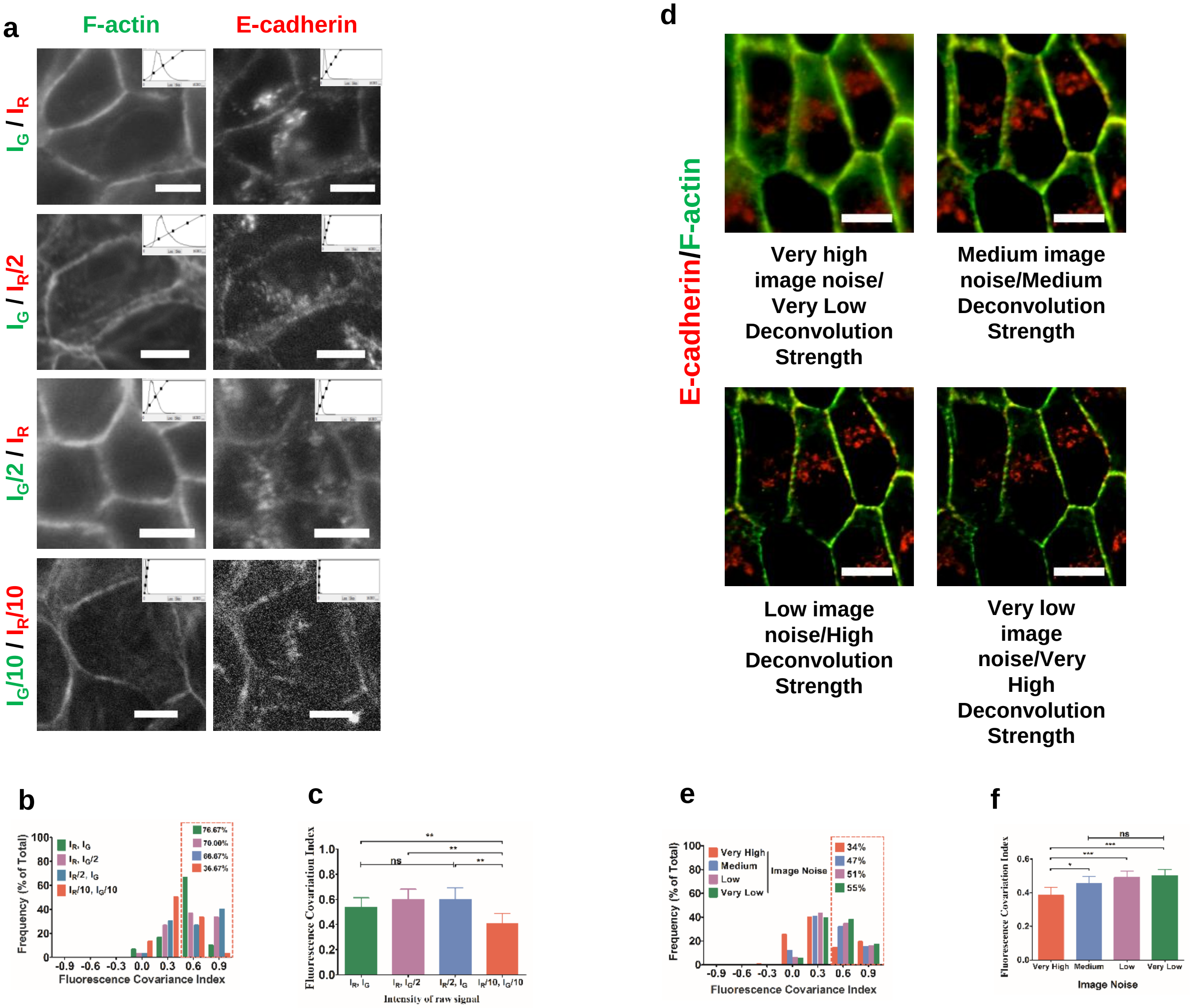

FCI measurements show little variation over images acquired with a wide range of SNRs. (a) Images representing MDCK cells in steady state immunostained for E-cadherin (Red) and F-actin (Green). Images were acquired using different exposure times **Top:Bottom** – 50ms (green), 200ms (red); 50ms (green), 100ms (red); 25ms (green), 200ms (red); 5ms (green), 20ms (red). Each image was contrast adjusted such that 1% of all pixels fell to the highest value while another 1% fell into the lowest value. The insets show the histograms for the intensity distribution and the line corresponding to the contrast adjustment's highest and lowest gray level. **(b)** Frequency distributions of FCI values for E-cadherin and F-actin with images acquired using different exposure times. Red box indicates the percentage of medium and high FCI values (0.5 – 1.0) for each case and bin width = 0.3. **(c)** Student's t-test was performed for individual pairs of data: I_R (red intensity = E-cadherin), I_G (green intensity = F-actin) was not significantly different from either one channels' exposure time being reduced by half; $I_R/10$, $I_G/10$ however had significantly lower FCI (I_R, I_G: $p = 0.0087$; I_R, $I_G/2$: $p = 0.0020$; $I_R/2$, I_G: $p = 0.018$) compared to each of the other exposure times. Error bars represent mean ± 95% CI. n = 30 cells per set of exposure times. **(d)** MDCK cells in steady state stained for E-cadherin (red) and F-actin (green) processed with varying deconvolution settings. **(e)** Frequency distributions of FCI values for E-cadherin and F-actin with different deconvolution strength settings. Red box indicates the percentage of medium and high FCI values (0.5 – 1.0) for each case and bin width = 0.3. **(f)** Mean FCI values for E-cadherin and F-actin at steady state where images were processed with varying deconvolution parameter for image noise. The result of a non-parametric Kruskal-Wallis test gives a p value < 0.0001 and the results of Dunn's post-hoc multiple comparison test are indicated on the graph (*** p < 0.001, *p < 0.1, ns: not significant). n = 150 for each setting.

Supplementary Figure 5

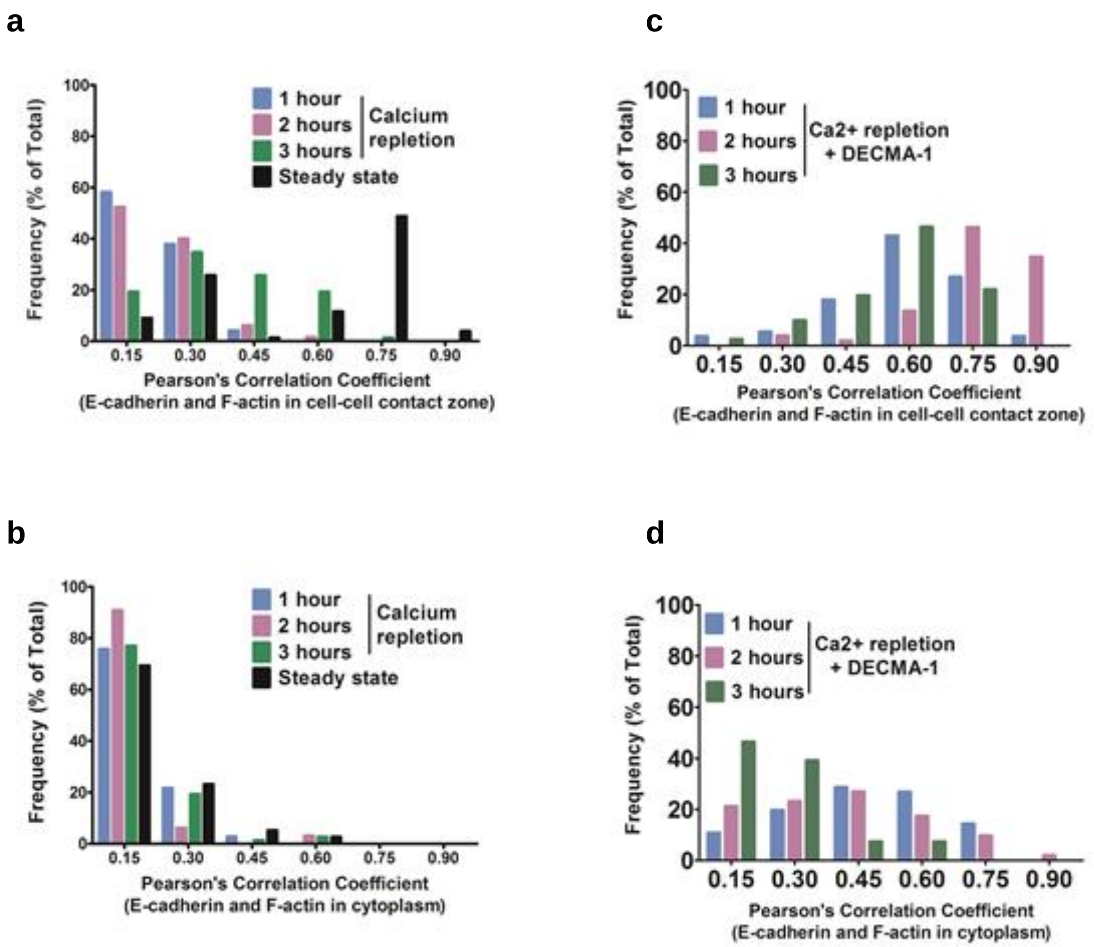

PCC values of F-actin and E-cadherin are sensitive to E-cadherin function and serve as reliable measures of adherens junction complex assembly. (**a, b**) Frequency distributions of PCCs with bin size of 0.15 for E-cadherin and F-actin: (**a**) at cell-cell contacts and, (**b**) in the cytoplasm. N (sample size) values: 1 hour = 74, 2 hours = 65 and 3 hours = 78. (**c, d**) Frequency distributions of PCCs with bin size of 0.15 for E-cadherin and F-actin: (**c**) at cell-cell contacts and (**d**) in the cytoplasm. N (sample size) values: 1 hour = 56, 2 hours = 52 and 3 hours = 41.

Supplementary Figure 6

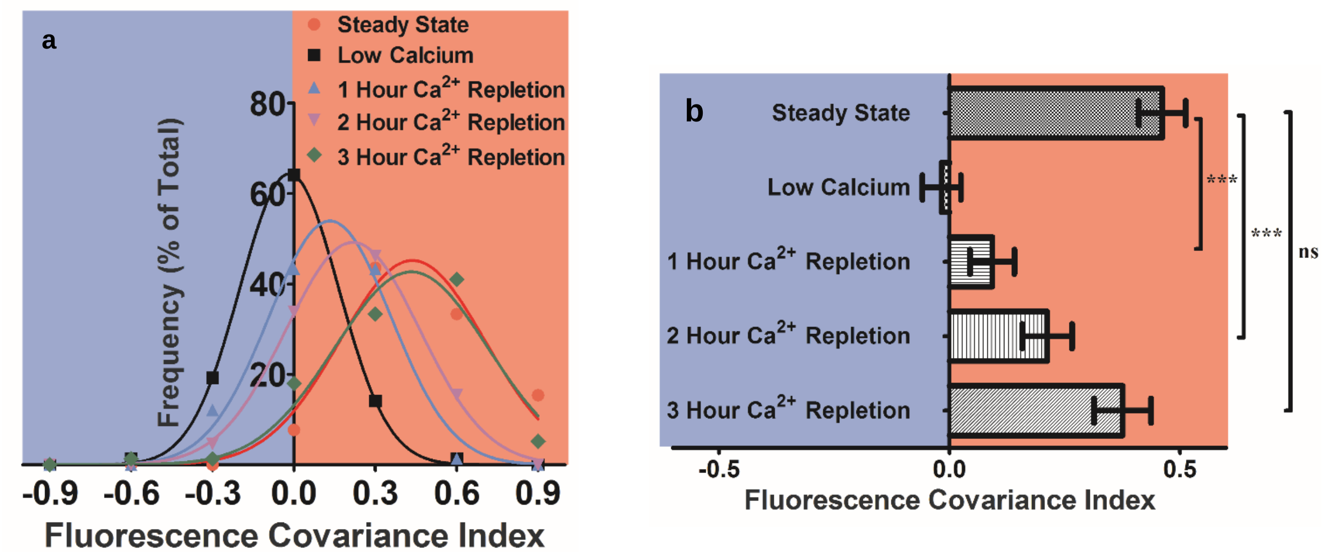

Adherens junction assembly/disassembly dynamics can be quantified using FCI analysis of E-cadherin and F-actin. (a) Curves represent the Gaussian best fits of the frequency distributions of FCI values for E-cadherin and F-actin following calcium repletion. R^2 values: steady state: 0.9575, low calcium: 0.9994, after calcium repletion: 1 hour: 0.9808, 2 hours: 0.9988, 3 hours: 0.9371. **(b)** Changes in FCI values for E-cadherin and F-actin during a calcium switch experiment. Bars represent mean ± 95% CI. A Kruskal-Wallis test (excluding low calcium data set) yielded a p value < 0.0001 and Dunn’s post-hoc multiple comparison test results are indicated on the graph (*** p < 0.001, ns: not significant).

Supplementary Figure 7

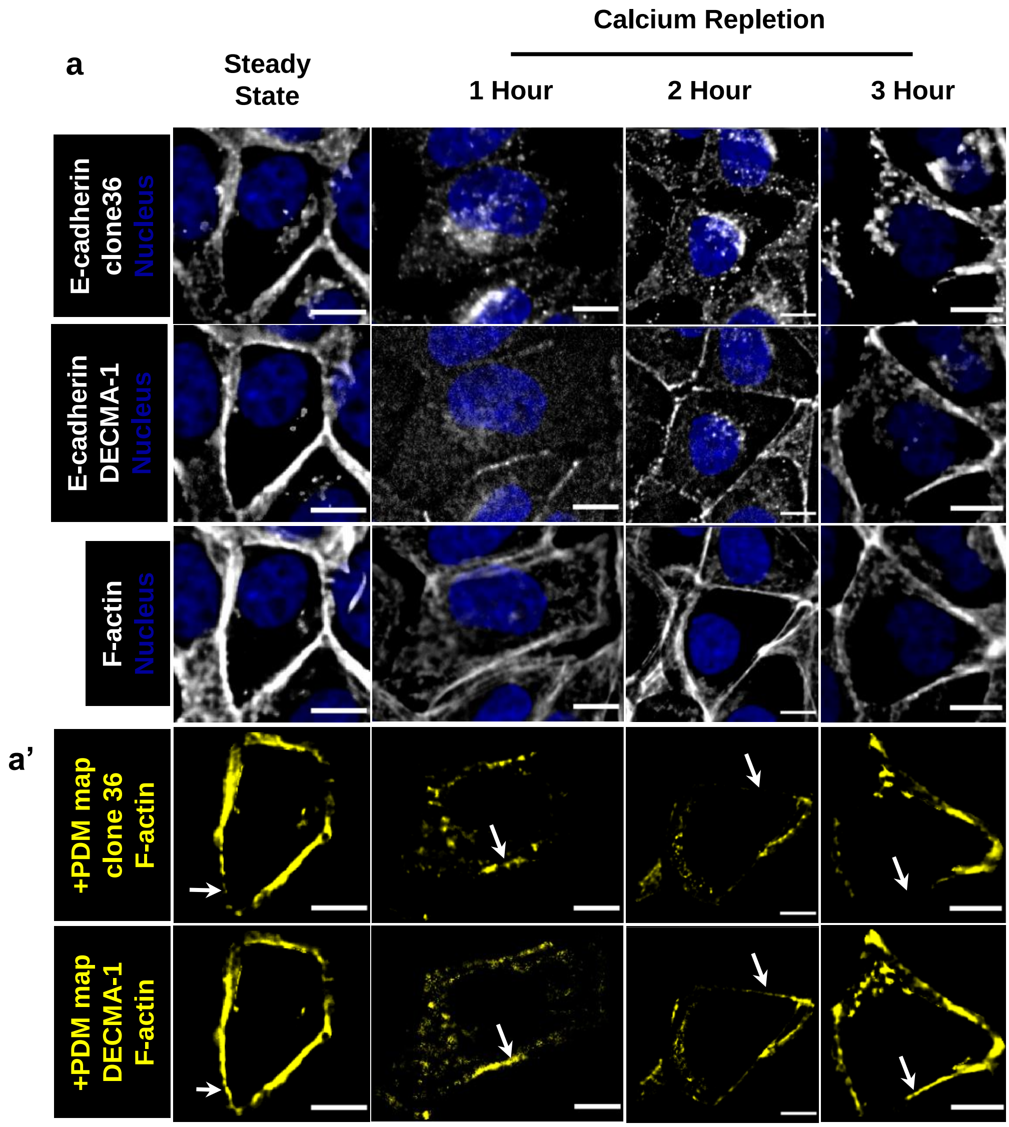

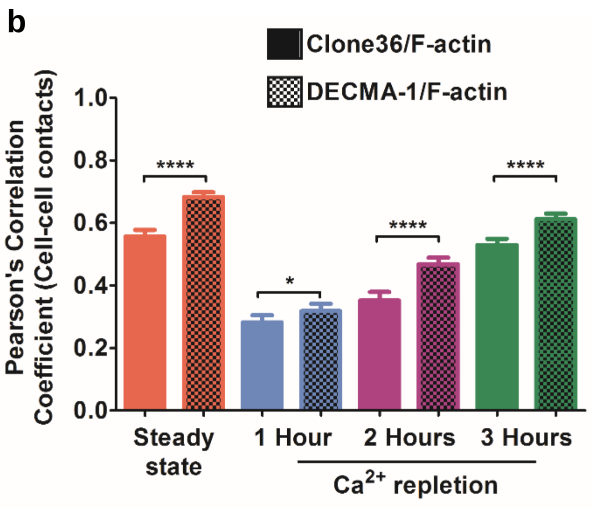

E-cadherin isoform specific antibodies show strong covariance following *de novo* cell-cell contact. (a) Left to right: Images representing MDCK cells in steady state; and 1 hour, 2 hours and 3 hours after calcium repletion. **Top to bottom**: Immunostained for E-cadherin clone36, E-cadherin DECMA-1 and F-actin. **(a')** +PDM maps for images shown in **(a)**. **Top panel**: E-cadherin clone36 : F-actin. **Bottom panel**: E-cadherin DECMA-1 : F-actin. White arrows point to areas with low signal for clone 36, and correspondingly a low covariance with F-actin. **(b)** Changes of FCI values for E-cadherin clone36 and F-actin. Solid bars. Changes of FCI values for E-cadherin DECMA-1 and F-actin during a calcium switch experiment. Checkerboard pattern. Steady state (n=123), 1 hour (n=113), 2 hours (n=115) and 3 hours (n=120). Independent student's t-test witch Welch's correction was performed for individual pairs of data (* $p < 0.05$, **** $p < 0.0001$). Error bars represent mean ± 95% CI.

Supplementary Figure 8

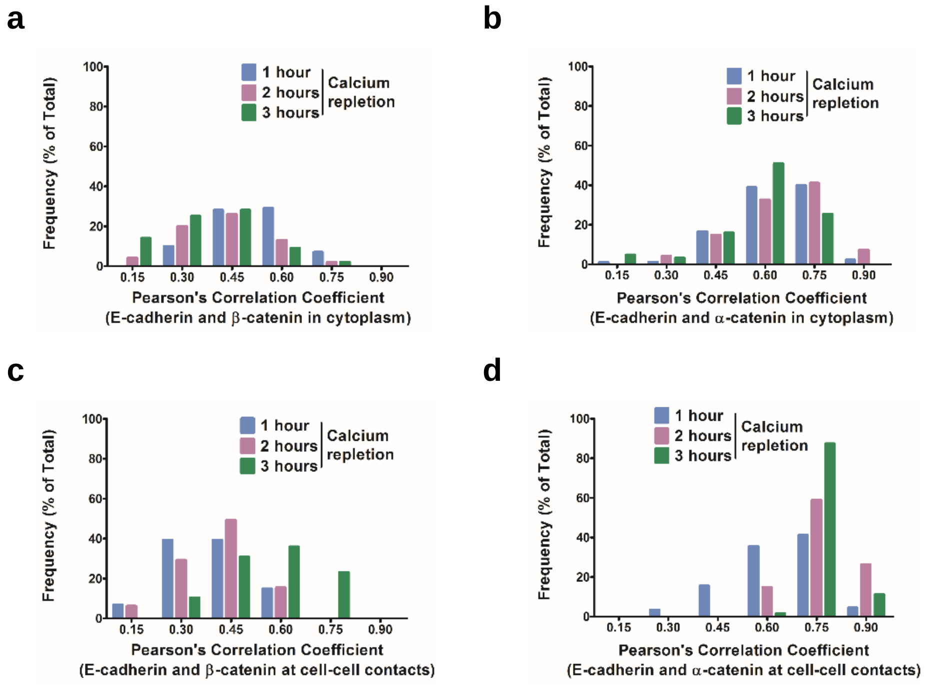

The minimal cadherin-catenin complex assembles in the cytoplasm and gets transported to the cell-cell contact. Frequency distributions of PCC values for: **(a)** β-catenin and E-cadherin in the cytoplasm, **(b)** α-catenin and E-cadherin in the cytoplasm, **(c)** β-catenin and E-cadherin cell-cell contacts and **(d)** α-catenin and E-cadherin cell-cell contacts. Bin width = 0.15. Note the PCC values in the cytoplasm for the cadherin-catenin complexes decrease, while the PCC values at cell-cell contacts increase, indicating that these complexes are assembled in the cytoplasm and trafficked to the cell-cell contacts.

Supplementary Figure 9

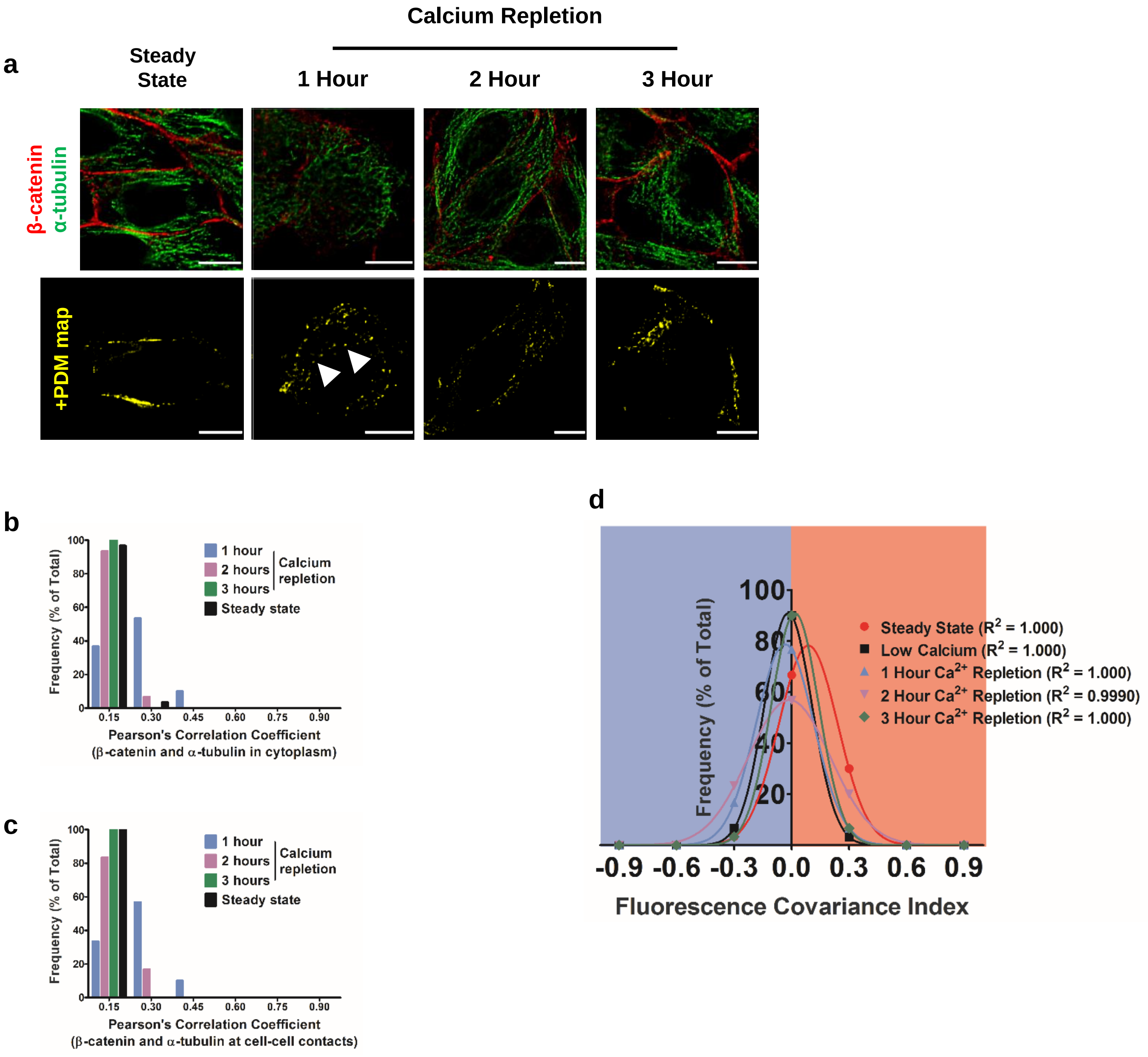

α-tubulin and β-catenin show different spatio-temporal association compared to cadherin and F-actin interactions during adherens junction assembly. (a) Top panel: MDCK cells in steady state, 1, 2 and 3 hours after calcium repletion fixed and immunostained for α-tubulin (green) and β-catenin (red). Scale bar = 10μm. **Bottom panel:** +PDM maps for the images shown in the top panel. Arrows in 1 hour calcium repletion indicate punctate positive correlations in the cytoplasm. Frequency distributions of PCC values for β-catenin and α-tubulin in: **(b)** cytoplasm zone and **(c)** cell-cell contact, during a calcium switch. n = 30 for each time point; Bin width = 0.15. **(d)** Gaussian best fit plots of FCI frequency distributions for α-tubulin and β-catenin during a calcium switch. Note all curves have a mean centered at zero, except steady state which has a slightly positive mean FCI value.

Supplementary Figure 10

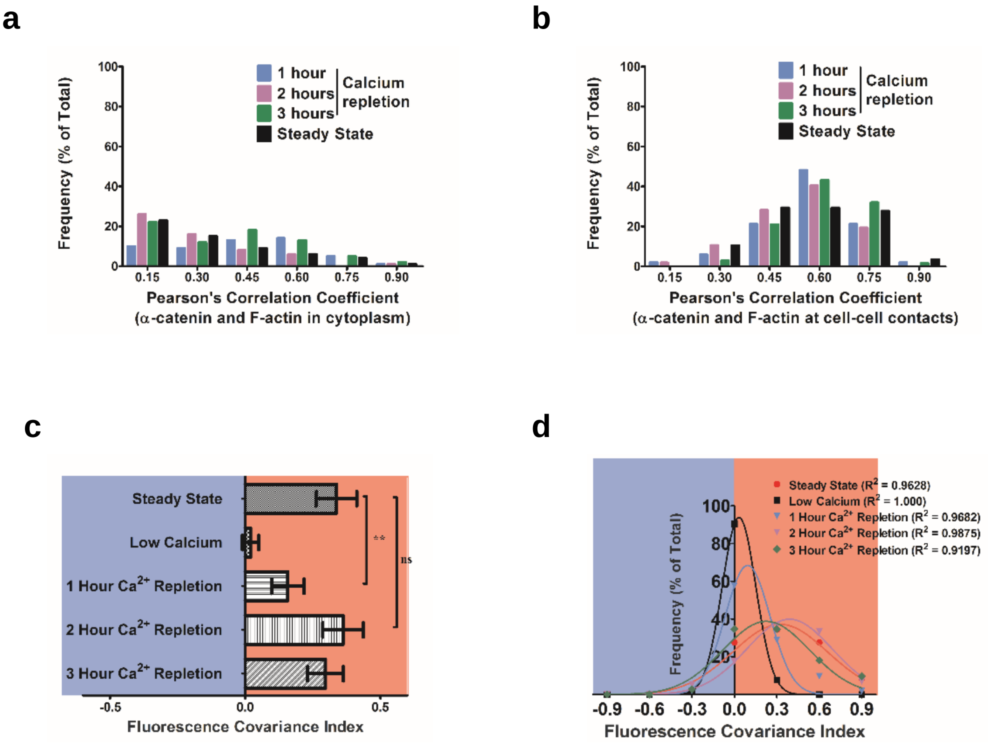

α-catenin and F-actin interaction can be used to indirectly quantify tissue tension profile. Frequency distributions of PCC values for α-catenin and F-actin: **(a)** in the cytoplasm and **(b)** at cell-cell contacts, during a calcium switch experiment. Bin width = 0.15. **(c)** Changes in FCI values for α-catenin and F-actin during a calcium switch. Error bars represent mean±95% CI. The result of a non-parametric Kruskal-Wallis test (excluding low calcium data set) gives a p value = 0.0003. The results of Dunn's post-hoc multiple comparison test are indicated on the graph (*** $p < 0.001$, ns: not significant). **(d)** Curves represent the Gaussian best fits for the frequency distributions for FCI values with a bin width of 0.3.

Supplementary Figure 10

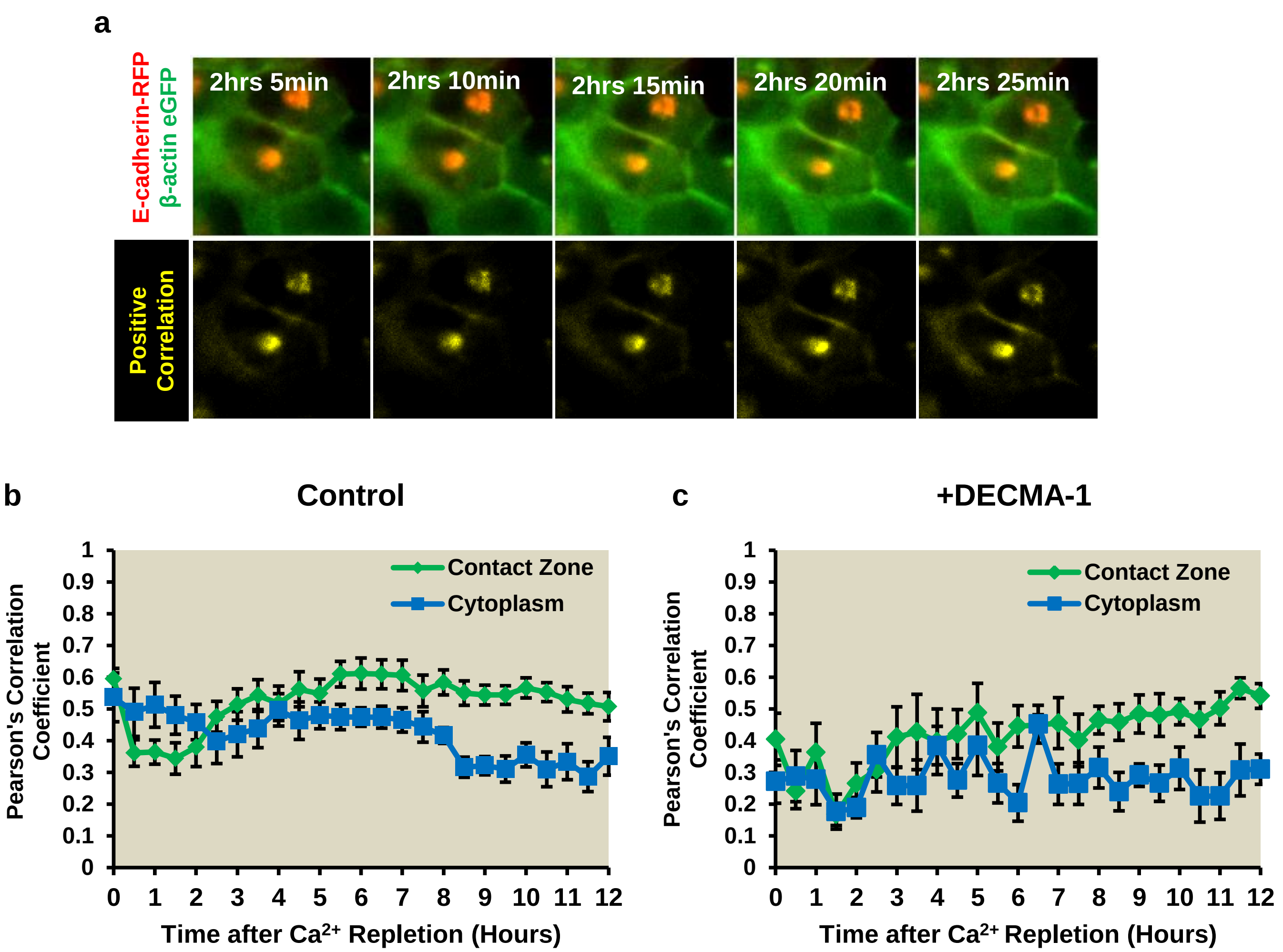

Complex formation in live cells expressing proteins with fluorescent fusion tags can be measured with 5 minute temporal resolution using live cell FCI analysis. (a) Top panel: Montage of live cell movie (2 hours – 2.5 hours) showing MDCK cells expressing E-cadherin-RFP (red) and β-actin-eGFP (green) after calcium repletion. **Bottom panel:** +PDM maps of images from the top panel. PCCs for β-actin-eGFP and E-cadherin-RFP in the contact zone and cytoplasm plotted every 0.5 hours following: **(b)** calcium repletion and **(c)** calcium repletion with E-cadherin function blocking antibody (DECMA-1). Error bars represent mean±s.e.m.
